# Cancer: Relief of Pain and Possible Cure

**Published:** 1908-12

**Authors:** 


**Cancer: Relief of Pain and Possible Cure.** By SKENE KEITH, M.B., and GEORGE E. KEITH, M.B., C.M. Pp. ix., 155. London: Adam and Charles Black. 1908.

This little book has been written to advocate the use of hypodermic injections containing iodipin, arseniate of iron, cacodylate of iron, and cinnamate of sodium. Numerous cases are recorded to show that benefit follows their use, both in relief of pain, improvement in the general condition of the patient, and diminution in the growth. Most of these are cases of breast cancer, but there are few of sarcoma. We noticed one case of disappearance of the growth, but occasionally malignant growth spontaneously disappears. The method rather reminds one of a prescription written by a doctor who has little faith in the value of any one drug for the cure of his patient's ailment, and therefore combines all he has ever heard of at all likely to do any good, in the hope that one or the other may do some good, but in what way and by what means he hardly knows. Perhaps the relief of pain in the cases treated by the author is due to the mental effect of the injection, and the improvement in general condition due to hope of ease in the minds of the patients. If the injections can really cause a decrease in the cancerous growth, why can they not remove the tumour in time in a certain proportion of the cases, as Coley's fluid does in sarcoma? Most of the cases treated have been inoperable ones. We do not like the suggestion of the authors to treat very early cases for some weeks by this method before resorting to operation, as we think no time should be wasted on any unproved remedy in such cases. No doubt such cases would be good test cases for this remedy, but in the interest of the individual patients we should strongly condemn such treatment.

